# Distinct Microbial Taxa Are Associated with LDL-Cholesterol Reduction after 12 Weeks of *Lactobacillus plantarum* Intake in Mild Hypercholesterolemia: Results of a Randomized Controlled Study

**DOI:** 10.1007/s12602-023-10191-2

**Published:** 2023-11-28

**Authors:** Felix Kerlikowsky, Mattea Müller, Theresa Greupner, Lena Amend, Till Strowig, Andreas Hahn

**Affiliations:** 1 Institute of Food Science and Human Nutrition, Leibniz University Hannover, 30167 Hannover, Germany; 2 Department of Microbial Immune Regulation, Helmholtz Center for Infection Research, Brunswick, Germany; 3 Cluster of Excellence RESIST (EXC 2155, Hannover Medical School, Hannover, Germany; 4 Center for Individualized Infection Medicine, Hannover, Germany

**Keywords:** Gut microbiota, Low-density lipoprotein, Dyslipidemia, Probiotic

## Abstract

Probiotic microbes such as *Lactobacillus* may reduce serum total cholesterol (TC) and low-density lipoprotein (LDL) cholesterol. The objective of this study was to assess the effect of *Lactobacillus plantarum* strains CECT7527, CECT7528, and CECT7529 (LP) on the serum lipids, cardiovascular parameters, and fecal gut microbiota composition in patients with mild hypercholesterolemia. A randomized, double-blinded, placebo-controlled clinical trial with 86 healthy adult participants with untreated elevated LDL cholesterol ≥ 160 mg/dl was conducted. Participants were randomly allocated to either placebo or LP (1.2 × 10^9^ CFU/d) for 12 weeks. LDL, HDL, TC, and triglycerides (TG), cardiovascular parameters (blood pressure, arterial stiffness), and fecal gut microbiota composition (16S rRNA gene sequencing) were assessed at baseline and after 12 weeks. Both groups were comparable regarding age, sex, and LDL-C at baseline. LDL-C decreased (mean decrease − 6.6 mg/dl ±  − 14.0 mg/dl, *P*_time*group_ = 0.006) in the LP group but not in the placebo group. No effects were observed on HDL, TG, or cardiovascular parameters or overall gut microbiota composition. Responders to LP intervention (> 5% LDL-C reduction) were characterized by higher BMI, pronounced TC reduction, higher abundance of fecal *Roseburia*, and lower abundance of *Oscillibacter*. In conclusion, 12 weeks of *L. plantarum* intake moderately reduced LDL-C and TC as compared to placebo. LDL-C-lowering efficacy of *L. plantarum* strains may potentially be dependent on individual difference in the gut microbiota. Trial registration: DRKS00020384, dated 07/01/2020.

**Supplementary Information:**

The online version contains supplementary material available at 10.1007/s12602-023-10191-2.

## Introduction

Cardiovascular diseases (CVD) are the leading cause of death worldwide [1]. The WHO estimated that more than 17.9 million peopled died from CVD in 2019. The most common form of CVD is coronary heart diseases caused by atherosclerosis. Epidemiological studies consistently show that increased plasma cholesterol and mainly the low-density lipoprotein cholesterol (LDL-C) fraction are associated with a high risk of developing atherosclerosis and myocardial infarction [2]. In moderate hypercholesterolemia (i.e., LDL-C level of ≥ 160 mg/dl– ≤ 200 mg/dl) and absence of CVD risk factors (e.g., smoking, hypertension, metabolic disorders), lifestyle modifications as nutritional adaptations can effectively reduce LDL-C level back to a normal range. The European Society of Cardiology (ESC) reported pharmacological intervention as the first choice of therapy for dyslipidemia if lifestyle modifications are not sufficient to reduce the atherosclerotic risk [3]. However, the desire for non-pharmacological intervention strategies is high, especially due to the side effects of statins affecting quality of life [3]. Among the nutritional modifications, probiotics have been implicated to beneficially modulate cholesterol metabolism. Probiotics are living microorganisms (e.g., *Lactobacillus* or *Bifidobacterium* spp.) that may colonize the gastrointestinal tract and confer beneficial health effects [4]. Consumption of probiotics mainly containing *Lactobacillus plantarum* and *Lactobacillus reuteri* species reduces circulating LDL-C concentrations in hypercholesteremic patients as shown in meta-analyses [5–7]. In vitro studies have suggested that the mechanism of action is based on the microbial expression of bile salt hydrolases (BSH), which are capable of deconjugating bile acids [8, 9]. Similar to the actions of pharmacological bile acid sequestrants, microbial deconjugation of bile acids interferes with recycling of bile, which stimulates the hepatic de novo bile acid synthesis and may ultimately lead to lower circulating LDL-C concentrations [9]. Other potential probiotic LDL-lowering mechanisms include incorporation of cholesterol in the microbial cell membranes or microbial metabolism of cholesterol to coprostanol [10]. Nonetheless, the beneficial effects of probiotics appear to be strain specific [11]. *Lactobacillus plantarum* CECT 7527, 7528, and 7529 strains have shown promising cholesterol-lowering efficacy in vitro [12] and in participants with dyslipidemia [13]. However, beneficial host effects of probiotics rely partly on at least transient colonization, which is mediated by the residing host commensal gut microbiota among other factors [14]. While there is little evidence that probiotics actually induce shifts in the overall community structure, multi-omics studies with single *Bifidobacteria* strains or probiotic mixtures show that the residential gut microbiota exerts functional and phylogenetic selection on the incoming probiotic bacteria [15, 16]. However, to date, no human study has yet investigated associations between features of the gut microbiota- and cholesterol-lowering effects after *Lactobacillus plantarum* CECT 7527, 7528, and 7529 intake. Hence, this randomized, placebo-controlled trial investigated the effect of 12-week probiotic supplementation with *Lactobacillus plantarum* (CECT 7527, 7528, and 7529 strains) on LDL-C and cholesterol metabolism in mildly hypercholesteremic participants (LDL-C ≥ 160 mg/dl and ≤ 220 mg/dl). To understand the response to the nutritional intervention at an individual level, we further investigated whether differences in LDL-C changes were associated with characteristics of the host’s resident gut microbiota.

## Methods

### Study Participants

In total, 86 healthy women and men between the age of ≥ 30 and ≤ 75 years and a body mass index (BMI) of ≥ 18 kg/m^2^ and ≤ 35 kg/m^2^ were recruited between January 2020 and December 2021 from the general population in the region of Hanover in Lower Saxony, Germany. Participants were included based on their fasting LDL-cholesterol level (LDL-C ≥ 160 mg/dl and ≤ 220 mg/dl) measured at the initial screening visit. Moderate LDL-hypercholesterolemia should be treated with lifestyle modifications in case of no concomitant risk factor for CVD. Relevant risk factors of CVD were defined as exclusion criteria: fasting triglycerides ≥ 220 mg/dl, BMI > 35 kg/m^2^, severe gastrointestinal or cardiovascular diseases, intake of immunosuppressive or chronic corticosteroids, or known allergy or intolerance to ingredients contained in the preparation. Further, we excluded subjects using lipid- and cholesterol-lowering drugs, taking dietary supplements that affect the lipid and cholesterol metabolism, regular intake of laxatives, and intake of antibiotics 3 months prior to the study. This study was approved by the Ethics Committee of the Medical Association of Lower Saxony (Hanover, Germany). The study is officially registered at the German Register of Clinical Studies (DRKS) with the identification number DRKS00020384 and was conducted in accordance with the guidelines of the Declaration of Helsinki (revised version, October 2008, Seoul, South Korea). Written informed consent was obtained from all participants.

### Study Design

This study was a double-blinded, randomized, placebo-controlled nutritional intervention trial. Participants with fasting LDL-C levels between ≥ 160 mg/dl and ≤ 220 mg/dl were randomly allocated to 12-week intake of either *Lactobacillus* mixture (“Lacto” group) or placebo capsules. *Lactobacillus* capsules contained 100 mg bacterial mixture containing 1.2 × 10^9^ CFU of *L*. *plantarum* CECT7527 (KABP011), *L*. *plantarum* CECT7528 (KABP012), and *L*. *plantarum* CECT7529 (KABP013) in portion 1:1:1 each; 340 mg maltodextrin; 0.5 mg silicon dioxide (release agent); and 95 mg capsule shell (hydroxypropyl cellulose, dyed with titanium dioxide). The preparation of Lactobacillus has been described elsewhere [17]. Placebo capsule contained 440 mg maltodextrin, 0.5 mg silicon dioxide, and 95 mg capsule shell (hydroxypropyl cellulose, dyed with titanium dioxide). The placebo capsule was matched to the *Lactobacillus* capsules for taste, color, and size. The sample size was *n* = 42 per group, calculated on the basis of observed change in a previous study using LDL-C variation as the primary outcome in a parallel group trial in hypercholesterolemic patients [13], estimating a moderate effect size of 0.3 and a significance level of 5% (two-sided) at a power of 80%. An additional 15% drop out rate was considered in the inclusion yielding a total *n* = 50 per group. The randomization was stratified by age and sex by an independent person otherwise not involved in the study. Both placebo and *Lactobacillus* strains were provided in identical looking capsules and packaging. Participants were instructed to ingest one capsule per day after a meal intake for 12 weeks. Before and after the intervention period, participants were invited for an examination day. During the intervention period, participants were asked to maintain their usual diet as well as physical activity habits. Compliance was ensured by counting the number of returned capsules after the 12-week intervention period.

### Screening and Examination Days

At the screening, participants were asked to come after an overnight fast (> 12 h) to the Institute of Food Science and Human Nutrition in Hanover. Eligibility criteria were assessed via a general health questionnaire and a rapid LDL-C test (Accutrend® Plus, Roche Diagnostics GmbH, Mannheim, Germany), where capillary blood drops were taken by a finger prick. Participants with fasting LDL-C concentrations between ≥ 160 mg/dl and ≤ 220 mg/dl were immediately included in the study, and the baseline examinations were conducted. Fasting blood samples from the antecubital vein were taken for further biochemical analyses. The baseline examination included measurement of body weight, body height, waist and hip circumference, blood pressure, and pulse. Body mass index was calculated by the ratio of weight to the squared height. Consequently, measurement of blood pressure and pulse were performed using volume plethysmography (boso ABI-system 100; BOSCH & SOHN, Germany) as previously described [18]. In short, after a 5-min rest in supine position, the systolic blood pressure at the left and right posterior on both sides was measured.

### Biochemical Blood Analysis

Fasting blood samples were collected in EDTA and serum monovettes (Sarstedt AG & Co., Nümbrecht, Germany). Blood samples were stored at 4 °C and were transferred on the same day to an accredited and certified laboratory (Laborärztliche Arbeitsgemeinschaft für Diagnostik und Rationalisierung e.V., Hannover, Germany). Triglycerides, LDL, and high-density lipoprotein cholesterol (HDL) were analyzed by a photometric method (Beckman Coulter GmbH, Krefeld, Germany). Total cholesterol and LDL/HDL-ratio were calculated from LDL and HDL values.

### Fecal Sample Collection

Stool samples were collected before the baseline and the final examination day after the intervention period at home using a fecal collection kit (Süsse Labortechnik, Gudensberg, Germany) and tubes containing 3.5 ml RNASepar stabilizer solution (Biosepar GmbH, Simbach am Inn, Germany). Upon arrival at the university, fecal samples were immediately stored at –80 °C. In addition, stool consistency was documented using the Bristol stool chart [19] in addition to the time of defecation and storage conditions.

### Gut Microbiota Sequencing

16S rRNA gene amplification of the V4 region (F515/R806) was performed according to an established protocol as previously described [20]. DNA isolation from stabilized fecal material was performed using the ZymoBIOMICS 96 MagBead DNA Kit (Freiburg, Germany) following the manufacturer’s instructions. Briefly, DNA was normalized to 25 ng/µl and used for sequencing PCR with unique 12-base Golary barcodes incorporated via specific primers (obtained from Sigma). PCR was performed using Q5 polymerase (New England Biolabs, New England Biolabs, Ipswich, Massachusetts) in triplicate for each sample, using PCR conditions of initial denaturation for 30 s at 98 °C, followed by 25 cycles (10 s at 98 °C, 20 s at 55 °C, and 20 s at 72 °C). After pooling and normalization to 10 nM, PCR amplicons were sequenced on an Illumina MiSeq platform via 250 bp paired-end sequencing (PE250). Using Usearch8.1 software package (http://www.drive5.com/usearch/), the resulting reads were assembled, filtered, and clustered. Sequences were filtered for low-quality reads and binned based on sample-specific barcodes using QIIME v1.8.0 [20]. Merging was performed using -fastq_mergepairs—with fastq_maxdiffs 30. Quality filtering was conducted with fastq_filter (-fastq_maxee 1), using a minimum read length of 250 bp and a minimum number of reads per sample = 1000. Reads were clustered into 97% ID OTUs by open-reference OTU picking, and representative sequences were determined by use of UPARSE algorithm [21]. Abundance filtering (OTU cluster > 0.5%) and taxonomic classification were performed using the RDP classifier executed at 80% bootstrap confidence cut off [22]. Sequences without matching reference dataset were assembled as de novo using UCLUST. Phylogenetic relationships between OTUs were determined using FastTree to the PyNAST alignment [23].

### Statistical Analysis

Normal distribution of the data was assessed by Shapiro–Wilk test and visual inspection. Non-parametric data were log-transformed to ensure normal distribution. To detect differences between the groups at baseline, Students *t*-test was used for normally distributed data and the chi-square test was applied for nominal variables. The intervention effect was determined using a repeated measures general linear model (GLM) with the within-subject factor time (t0, t12) and between-subject factor group (Lacto, placebo) comparison. *P*-values of < 0.05 were considered significant. Analysis of the clinical data was performed in SPSS (28.0.1.0 (142)).

Resulting OTU absolute abundance table and mapping file were used for statistical analyses using the package *phyloseq* [24]*.* Samples were rarefied to an even sequencing depth. Alpha diversity indices for Shannon’s index, inverse Simpson index, observed richness index, and Chao1 richness index were calculated, and a paired Wilcoxon signed-rank test was used comparing time points within each group. Barplots of the relative abundance of each individual were visualized using the *microViz* package [25]. Samples were filtered to at least 10% of prevalence of the total samples before analysis of differential abundances. Differential abundances were compared within groups before and after the intervention with the centered log-ratio-transformed abundances on phyla, family, and genus level using Wilcoxon signed-rank test with FDR adjustment for multiple testing as described. To detect compositional differences in the microbiota between groups after the intervention, permutational multivariate analysis of variance (PERMANOVA) was conducted using generalized weighted UniFrac distance as implemented in the package *vegan* [26] and *GUniFrac* [27]. The individual participant ID was used as block factor to account for repeated measures. Multidimensional scaling ordination was used to visualize clustering of samples based on generalized UniFrac distances using the *microViz* package. Ellipses were drawn based on the 95% confidence limit of the standard error of points for each participant. Multivariate association with linear models (MaAsLin2) [28] were used to investigate associations between taxa abundances, responder status, LDL-C, and total cholesterol changes. For further analyses within the intervention group, we identified subjects with an LDL-C reduction greater than 5% as responders and subjects with an LDL-C reduction ≤ 5% as non-responders, based on the expected effect of the supplement used in the intervention and also on clinical relevance for the prevention of coronary heart disease.

Differences of microbial taxa between responder and non-responder (as fixed factor) were controlled for age, sex, BMI, stool consistency, and participant ID (as random effects). Associations between microbial taxa, LDL-C, and total cholesterol concentrations (as fixed factor) were controlled for baseline LDL-C or total cholesterol concentrations, age, sex, BMI, stool consistency, and participant ID (as random effects). Default settings of MaAsLin2 were used, and *q*-values < 0.25 were considered significant. All microbiota statistical analyses were carried out in R (version 4.2.1).

## Results

### Baseline Characteristics

In total, 86 participants with mild hypercholesterolemia (LDL-C ≥ 160 mg/dl and ≤ 220 mg/dl) were included in this study. Of these, 83 participants provided a complete stool sample from both time points before and after the intervention for 16S rRNA gene sequencing (Supplemental Fig. S1). Participants in the Lacto group showed a good compliance as 90% ± 4% of capsules were consumed during the study period. There were no significant differences in age, BMI, fasting glucose, and blood pressure at baseline between both groups (Table 1).

**Table 1:** Baseline characteristics of the study participants

**Variables**	**Lacto (*n*** **= 43)**	**Placebo (*n*** **= 43)**	***P***
Sex (*male/female*)	14/34	14/30	0.631^a^
Age (*y*)	63.6 ± 7.0	63.3 ± 7.9	0.690
Weight (*kg*)	75.1 ± 13.9	76.7 ± 13.5	0.681
Body mass index (*kg/m*^*2*^)	26.4 ± 4.2	26.2 ± 3.9	0.724
Fasting glucose (*mmol/L*)	5.6 ± 0.6	5.5 ± 0.4	0.856
Waist:hip ratio	0.87 ± 0.09	0.84 ± 0.09	0.273
Systolic blood pressure (*mmHg*)	141 ± 15	138 ± 12	0.243
Diastolic blood pressure (*mmHg*)	88 ± 8	86 ± 7	0.429

Data are mean ± SD. Group differences were assessed using independent Student’s *t*-test

*LDL* low-density lipoprotein

^a^Group differences in sex were assessed using Chi^2^ test

### Lipid Metabolism after Lactobacillus plantarum Intake 

We observed a significant reduction of LDL-C concentrations in the Lacto group compared to the placebo group (mean LDL-C change: Lacto group, − 6.6 mg/dl ±  − 14.0 mg/dl; placebo group, 2.3 mg/dl ± 13.9 mg/dl; *P* = 0.006 for the time*group difference, Table 2), while there were no significant changes in the placebo group after the intervention. Further, total cholesterol was significantly reduced in the Lacto group (mean total cholesterol decrease − 10.4 mg/dl ± 24.2 mg/dl, *P* = 0.045, Table 2) when compared to the placebo group. We observed no differences in triglycerides, HDL-C concentrations, and LDL:HDL ratio after the intervention period between both groups.

**Table 2:** Blood lipid parameters before and after the intervention period

**Variables**		**Lacto (*n* = 43)**	**Placebo (*n* = 43)**	***P***
LDL cholesterol (*mg/dl*)	Pre	190 ± 19.4	188 ± 20.6	0.006*
	Post	184 ± 21.2	190 ± 21.1	
Total cholesterol (*mg/dl*)	Pre	280 ± 32.3	282 ± 32.4	0.045*
	Post	269 ± 36.5	282 ± 35.7	
HDL cholesterol (*mg/dl*)	Pre	64.2 ± 15.1	64.9 ± 14.5	0.879
	Post	64.1 ± 16.5	64.5 ± 14.6	
Triglycerides, *mg/dl*	Pre	114.2 ± 37.0	135.4 ± 54.5	0.775
	Post	117.2 ± 39.9	136.5 ± 60.0	
LDL:HDL ratio	Pre	3.1 ± 0.7	3.0 ± 0.7	0.133
	Post	3.0 ± 0.6	3.1 ± 0.6	

Data are mean ± SD and analyzed using generalized linear models with time (pre/post) and group as fixed factors

*LDL* low-density lipoprotein, *HDL* high-density lipoprotein

^*^P-value represents time*group interaction

### Responder and Non-responder Observation in Clinical Data

We observed great interindividual variation in LDL-C response after 12 weeks of *Lactobacillus* intake (Supplemental Fig. S2). Therefore, we classified participants in the Lacto group into responder (i.e., > 5% LDL-C decrease after intervention, *n* = 20) and non-responder (i.e., ≤ 5% LDL-C decrease or no change after intervention, *n* = 23). The lipid profile of responders was similar to non-responders, yet responders had a higher body weight (79.9 kg ± 11.7 kg in responder vs. 71.6 kg ± 14.1 kg in non-responder, *P* = 0.044) and BMI (28.1 kg/m^2^ ± 4.5 kg/m^2^ in responder vs. 25.2 kg/m^2^ ± 3.9 kg/m^2^ in non-responder, *P* = 0.029, Supplemental Table S1) at baseline. Responders had a significantly higher reduction in total cholesterol concentrations after the intervention when compared to non-responders (Supplemental Table S1), while fasting triglycerides, HDL-C, and LDL:HDL ratio were not significantly different between responder and non-responder in the Lacto group.

### Gut Microbiota Composition after Lactobacillus plantarum Intake

**Fig. 1:**
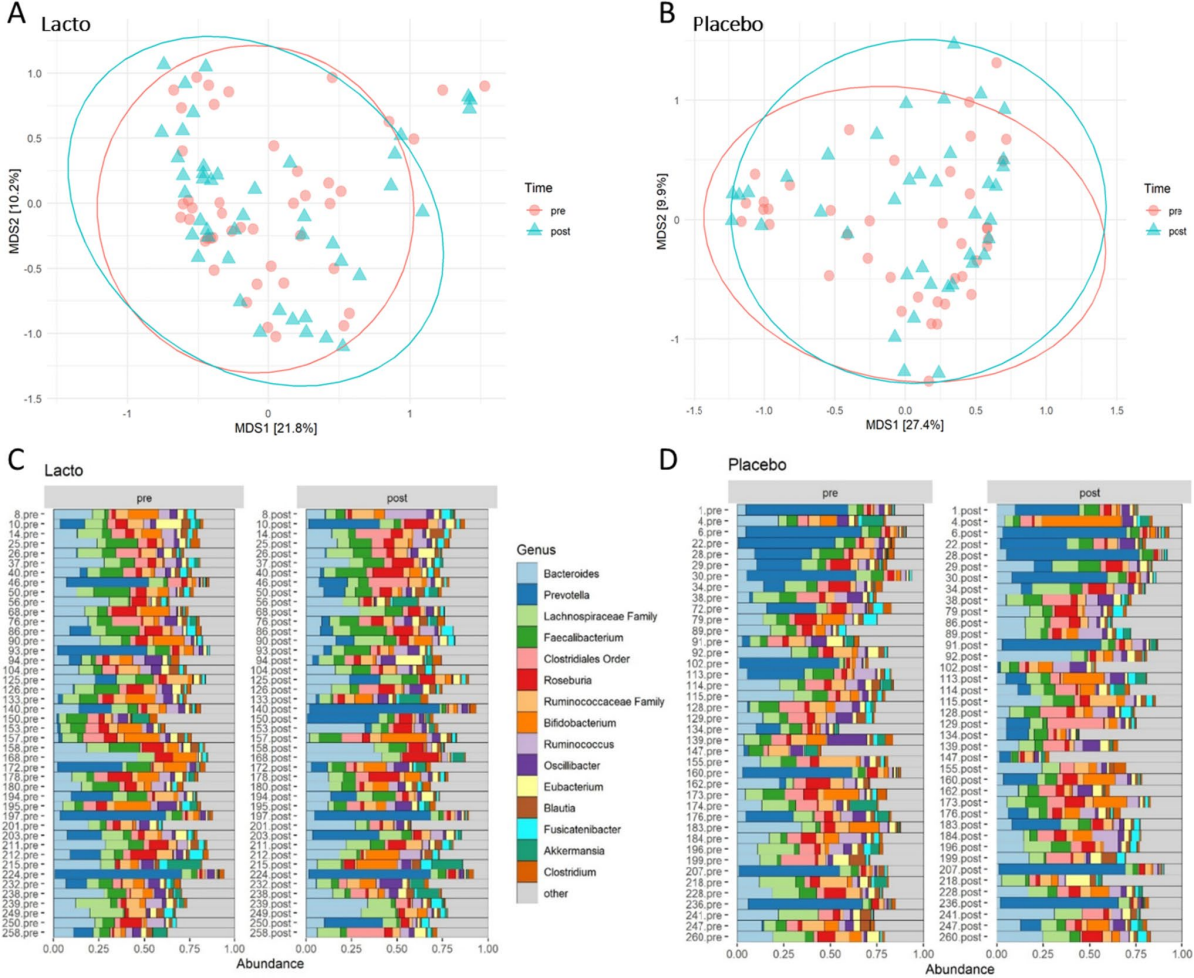
Gut microbiota composition in the Lacto (*n* = 43) and placebo groups (*n* = 43) before (pre) and after (post) the intervention. Multidimensional scaling (MDS) of generalized UniFrac distances between the gut microbiotas of the Lacto group (**a**) and placebo group (**b**) color-coded by time points (PERMANOVA (genus level) time*group *P* > 0.05). Ellipses represent 95% confidence intervals around the centroid of each time point. Bar plots show the top 15 taxa at genus level at both time points per individual in the Lacto (**c**) and placebo groups (**d**). Numbers in the labels represent participant ID

In total, 16S rRNA gene sequencing of fecal samples was analyzed in 43 participants of the Lacto group and 40 participants of the placebo group. A total of 2,976,226 reads with a mean of 17,715 reads of the V4 region of the 16S rRNA gene were obtained. The alpha diversity indices were not significantly different in the Lacto group as compared to the placebo group after the intervention (paired Wilcoxon *P* > 0.5, Supplemental Fig. S3). There were no major taxonomic compositional changes of the gut microbiota between the groups after the intervention period as assessed using generalized weighted UniFrac distances (PERMANOVA *P* time*group > 0.5, Fig. 1a, b). When comparing differential abundances on phylum, family, or genus level before and after the intervention, no taxa were significantly different (paired Wilcoxon FDR-adjusted *P* > 0.05) within the groups (Fig. 1c, d and Supplemental Table S1). *Lactobacillus* abundance at genus level was not significantly different at baseline or after the intervention between the groups (Supplementary Fig. S4).

### Difference in Gut Microbiota in Responder vs. Non-responder

**Fig. 2:**
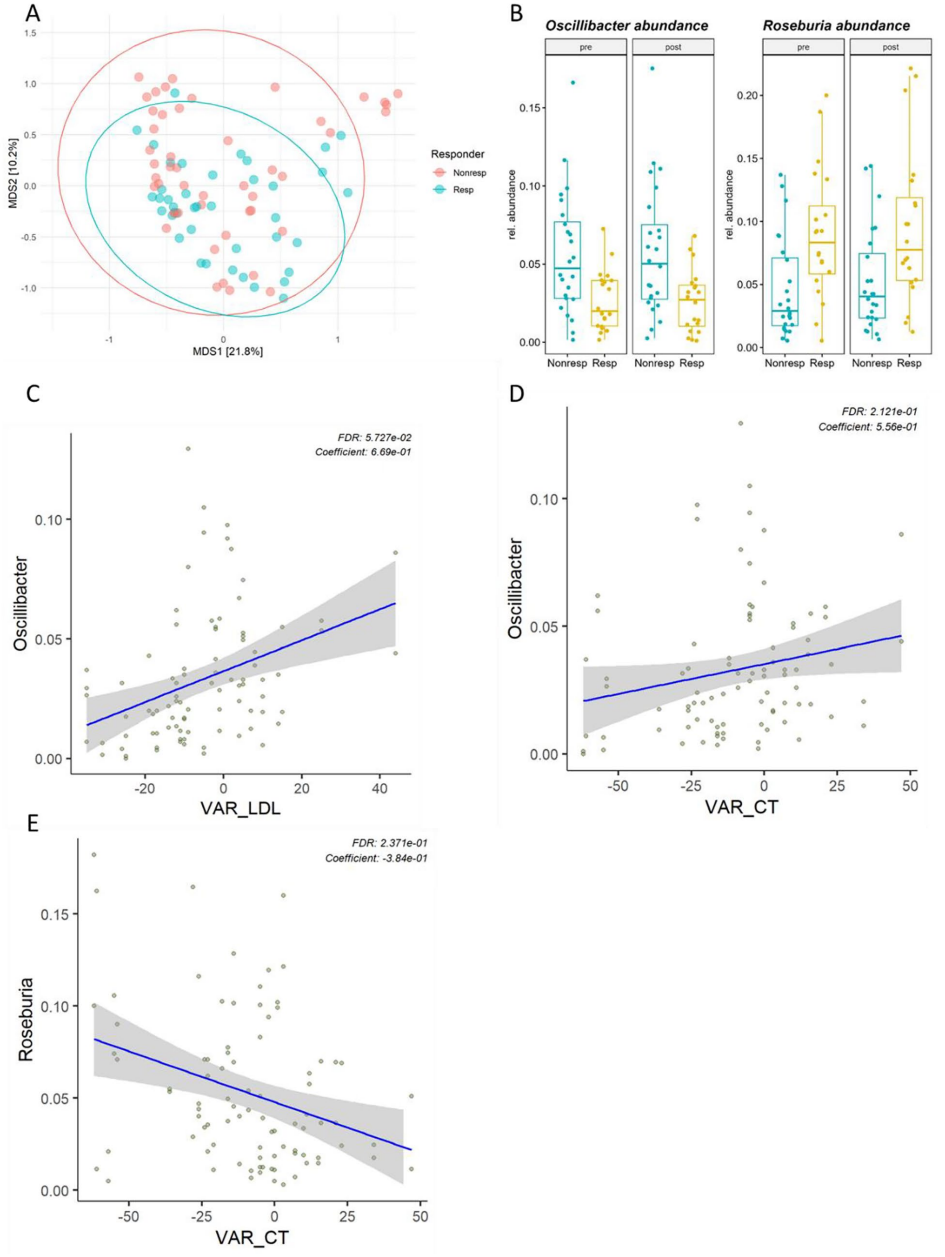
Gut microbiota difference in responder vs. non-responder and association with clinical parameters. **a** Multidimensional scaling (MDS) of generalized UniFrac distances between the gut microbiota of responder vs. non-responder (PERMANOVA (genus level), responder *R*^2^ = 9%, *P* = 0.03). Ellipses represent 95% confidence intervals. **b** Box plots showing the relative abundance of *Roseburia* and *Oscillibacter* in responders and non-responders before and after the intervention. Scatter plots of changes (post vs. pre) of LDL-C (**c**) and total cholesterol (**d**, **e**) and the relative abundance of *Oscillibacter* and *Roseburia* on both time points after adjusting for covariates (baseline LDL-C or total cholesterol, respectively, age, sex, BMI, stool consistency, participant ID). FDR-adjusted *P*-values from MaAsLin2 models and coefficients are shown in each panel. Resp, responder; Nonresp, non-responder; VAR_LDL, change in LDL-C in mg/dl compared to baseline; VAR_CT, change in total cholesterol in mg/dl compared to baseline

Further, we investigated differences in the gut microbiota composition of responders and non-responders in the Lacto group. Alpha diversity indices were not different between responders and non-responders after *Lactobacillus plantarum* intake (Supplemental Fig. S5). Further, we did not observe differences in beta diversity indices using generalized weighted UniFrac distances over time (PERMANOVA, time*responder, *P* > 0.5). However, the gut microbiota composition of responder was significantly different to the gut microbiota composition of non-responders independent of the intervention (PERMANOVA responder *P* = 0.03, Fig. 2a). Using multivariate linear models (MaAsLin2) with the covariates age, sex, BMI, participant ID, and stool consistency, responders had consistently higher relative abundance of the *Roseburia* (MaAsLin2 coeff 1.01,* q* = 0.05) and lower abundances of *Oscillibacter* (MaAsLin2 coeff 1.36,* q* = 0.05) on genus level independent of the intervention as compared to non-responders (Fig. 2b and Supplemental Table S3). To confirm these associations, we further investigated whether changes of LDL-C and total cholesterol concentration as continuous variables were associated with these differential abundant taxa. Higher relative abundances of *Oscillibacter* were both associated with higher LDL-C and higher total cholesterol concentrations after the intervention in the Lacto group using multivariate models adjusted for baseline LDL-C or total cholesterol, respectively, age, sex, BMI, participant ID, and stool consistency (Fig. 2c, d and Supplemental Table S3). Conversely, lower concentrations of total cholesterol but not LDL-C after the *Lactobacillus plantarum* intake were associated with higher *Roseburia* abundance (Fig. 2e and Supplemental Table S3).

## Discussion

The primary aim of this randomized, placebo-controlled study was to investigate the effect of 12-week intake of *Lactobacillus plantarum* strains CECT7527, CECT7528, and CECT7529 on LDL-C concentrations in patients with mild hypercholesterolemia (≥ 160 mg/dl LDL-C). A secondary aim was the investigation of associations between the LDL-C response and the gut microbiota composition.

In contrast to a previous intervention study with *L. plantarum* strains CECT7527, CECT7528, and CECT7529 in patients with hypercholesterolemia [13], we observed a moderate but significant decrease of − 3.2% in LDL-C after 12-week supplementation of *L. plantarum* strains CECT7527, CECT7528, and CECT7529. Remarkably, the reduction in total cholesterol (− 3.3%) was less pronounced in the present study as compared to a reduction of − 13% in total cholesterol as reported previously [13]. However, differences in cholesterol metabolism may partly account for the observed differential study outcomes, as the dyslipidemia both in LDL-C and total cholesterol was more severe in the present study population as compared to the previous study [13]. Even though the observed reduction of LDL-C and cholesterol is relatively minor, a reduction of 1% of cholesterol may already lead to a 2–3% reduced risk of developing coronary heart disease [29]. A more recent meta-analysis suggested that a decrease of 10 mg/dl LCL-C reduced the relative risk of coronary heart diseases by 7.1% [16].

We did not detect differences in the abundance of *Lactobacillus* on genus level after the intervention, which may be explained by the sampling site as the gut microbiota sampled from feces rather resemble the composition in the distal colon [30]. Of note, successful colonization of probiotics has been observed in more proximal intestinal niches as well as along spatial gradients from gut mucosa to gut lumen [15, 30]. Besides, there is a clear microbial succession along the intestine [31]. Like the ripples of a wave fade as distance increases from the perturbation, microbiota could change in the ileum and remain totally unchanged in the distal colon. In addition, we did not observe any shifts in the overall microbial community after the intervention, thus resilience and stability of the residing gut microbiota might have hampered colonization of incoming, possibly non-native *Lactobacillus* strains [32]. The observed—though moderate—cholesterol-lowering effect may be in place even when *Lactobacillus plantarum* presence is only transient. Interestingly, there were great interindividual differences in LDL-C response between the participants; hence, we further explored differences in responder (> 5% LDL-C decrease) vs. non-responder (≤ 5% LDL-C or no change) in the Lacto group. Surprisingly, responder and non-responder did not differ with regard to their baseline LDL-C concentrations, total cholesterol, or triglyceride, thus ruling out the possibility that high initial cholesterol or triglyceride concentrations account for the observed differences LDL-C regarding reduction after *L. plantarum* intake as suggested previously [7]. In addition, responders had a significantly higher BMI when compared to non-responders. As elevated bile acids are frequently observed in overweight/obesity [33, 34], BSH activity of *Lactobacillus plantarum* strains may be more pronounced in the presence of bile acid abundancy in overweight/obese responders and hence promote cholesterol scavenging [9]. In addition, microbiota-mediated factors may be responsible for the observed interindividual differences of LDL-C lowering after probiotic intake as indicated previously [15]. The gut microbiota composition between responders and non-responders differed independent of the intervention period, after adjusting for BMI and other possible confounding factors. Responders had consistently higher fecal abundances of the *Roseburia*, a beneficial butyrate-producing gut commensal [35]*.* The abundant presence of *Roseburia* in responders vs. non-responders may constitute a favorable niche for the incoming *L. plantarum*, as *Roseburia* and *Lactobacillus* may interact through cross-feeding networks involving acetate and butyrate [36]*.* Moreover, responders had lower abundances of the gut commensal *Oscillibacter*, a putative butyrate and valerate producer [37], which was correlated with reductions in LDL-C and total cholesterol after the intervention. The latter supports previous findings reporting lower abundance of *Oscillibacter* in lean as compared to obese participants [38–40]. Of note, *Oscillibacter* presence has been negatively associated with HDL-C concentrations in an observational cohort of healthy and hypercholesteremic men [41]. Contrary, Mendelian randomization analyses describe a causal relationship of *Oscillibacter* abundance in feces and reduced plasma triglycerides in a large Chinese cohort [42]. However, due to the lack of mechanistic insights on *Oscillibacter*, it is difficult to set these controversial associations in a physiological context. To conclude, responders characterized by markedly reduced LDL-C and total cholesterol after *L. plantarum* intake differed to non-responder with regard to BMI, body weight, and abundance of *Roseburia* and *Oscillibacter*. Identification of predictive factors for response to appropriate nutritional intervention to reduce LDL-C concentrations may be an important contribution to future personalized nutritional strategies.

## Limitations

This study has several limitations. First, we did not monitor dietary intake before and after the intervention, which might have changed during the intervention period and thus influence clinical outcomes. Secondly, the study was conducted during two major COVID-19 lockdown periods in Germany (Jan 2020 to Dec 2021); thus, it is likely that dietary habits as well as physical activity levels changed during the study period. These factors could possibly influence the difference in efficacy between our study and a previously published one using the same strains [13]. Besides, 16S rRNA gene sequencing has limitations regarding its detection sensitivity on species or even strain level; however, more sensitive methods such as qPCR with strain specified primers were unfortunately not available in the present study.

## Conclusion

In conclusion, 12 weeks of *Lactobacillus plantarum* strain intake has a moderate effect on lowering LDL-C and cholesterol levels in mildly hypercholesterolemic patients. Even though transiently the LDL-lowering efficacy of the probiotic *L. plantarum* strains may be mediated by individual difference in the gut microbiota, we detected difference in *Oscillibacter* and *Roseburia* abundances in responder vs. non-responder. Thus, further studies should focus on elucidating the characteristics of the resident gut microbiota associated with *L. plantarum* intake to predictively and precisely achieve beneficial effects on lipid metabolism.

## Supplementary Information

Below is the link to the electronic supplementary material.

Supplementary file1 (DOCX 306 KB)

## Data Availability

Sequence data have been deposited at ENA (European Nucleotide Archive), under the accession number PRJEB57654.
