# Supplementary material for: Distinct Microbial Taxa Are Associated with LDL-Cholesterol Reduction after 12 Weeks of Lactobacillus plantarum Intake in Mild Hypercholesterolemia: Results of a Randomized Controlled Study
Source: Probiotics Antimicrob Proteins. 2023 Nov 28;17(3):1086–95. doi: 10.1007/s12602-023-10191-2 (PMC12055864; doi:10.1007/s12602-023-10191-2)
Supplement: Supplementary file 1 — Supplementary file1 (DOCX 306 KB) [file 12602_2023_10191_MOESM1_ESM.docx]

Supplemental of the manuscript:

“Distinct microbial taxa are associated with LDL-cholesterol reduction after 12 weeks of Lactobacillus plantarum intake in mild hypercholesterolemia” Results of a Randomized Controlled

Felix Kerlikowsky^1^*, Mattea Müller^1^*, Theresa Greupner^1^, Lena Amend^2,3^, Till Strowig^2,3,4^, Andreas Hahn^1^

^1^Institute of Food Science and Human Nutrition, Leibniz University Hannover, Hannover, Germany

^2^Department of Microbial Immune Regulation, Helmholtz Center for Infection Research, Braunschweig, Germany

^3^Cluster of Excellence RESIST (EXC 2155), Hannover Medical School, Hannover, Germany;

^4^Center for Individualized Infection Medicine, Hannover, Germany

Correspondence: Felix Kerlikowsky

Leibniz University Hannover

Institute of Food Science and Human Nutrition

30167 Hannover, Germany

Email: kerlikowsky@nutrition.uni-hannover.de

Tel.: +49 511 762 5430

Fax: +49 511 762 5729

**Supplemental Fig. 1** CONSORT flow diagram of the study


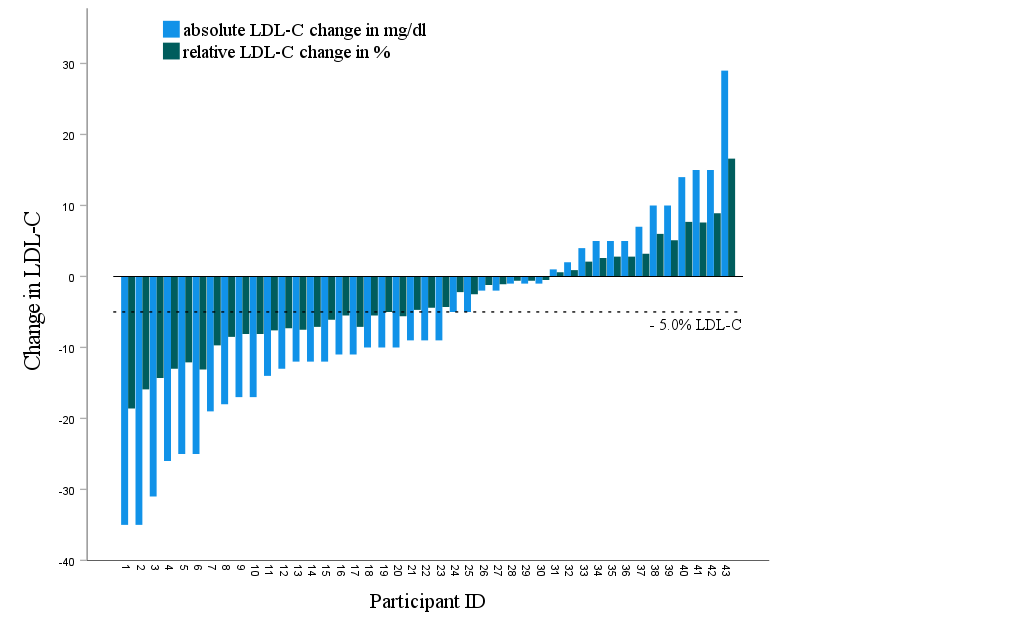


**Supplemental Fig. 2** Absolute and relative changes of LDL-C concentrations (n= 43) after 12 weeks of Lactobacillus plantarum intake

**Supplemental Table 1.** Baseline characteristics of responder and non-responder within the Lacto group

| **Variables** | **Responder (n=20)** | **Non-responder (n=23)** | ***P*** |
| --- | --- | --- | --- |
| Sex, male/female | 5/15 | 6/17 | 0.935 † |
| Age, *y* | 64.2 ± 6.6 | 64.7 ± 6.2 | 0.818 |
| Weight, *kg* | 79.9 ± 11.7 | 71.6 ± 14.1 | 0.044 |
| Body mass index, *kg/m^2^* | 28.1 ± 4.5 | 25.2 ± 3.9 | 0.029 |
| Waist:hip ratio | 0.88 ± 0.1 | 0.85 ± 0.09 | 0.343 |
| LDL cholesterol, *mg/dl* | 193.0 ± 18.1 | 188.5 ± 20.6 | 0.452 |
| ∆ LDL cholesterol , *mg/dl* | -18.1 ± 8.4 | 3.4 ± 9.2 | >0.001 |
| Total cholesterol, *mg/dl* | 280 ± 30 | 327 ± 43 | 0.967 |
| ∆ Total cholesterol, *mg/dl* | -28.45 ± 19.2 | 5.3 ±15.7 | >0.001 |
| HDL cholesterol, *mg/dl* | 60.8 ± 10.6 | 67.0 ± 17.8 | 0.174 |
| ∆ HDL cholesterol, *mg/dl* | -2.5 ± 8.9 | 1.9 ± 6.1 | 0.064 |
| Triglycerides, mg/dl | 128 ± 40.0 | 102 ± 30.1 | 0.020 |
| ∆ Triglycerides, mg/dl | -3.0 ± 35.6 | 8.2 ± 19.5 | 0.200 |
| LDL:HDL ratio | 3.2 ± 0.7 | 3.0 ± 0.7 | 0.246 |
| ∆ LDL:HDL ratio | -0.2 ± 0.5 | -0.1 ± 0.4 | 0.210 |

Values are given as means ± SD. Group differences were assessed using independent Student’s t-test between responder and non-responder. †Group differences in sex were assessed using chi-squared test


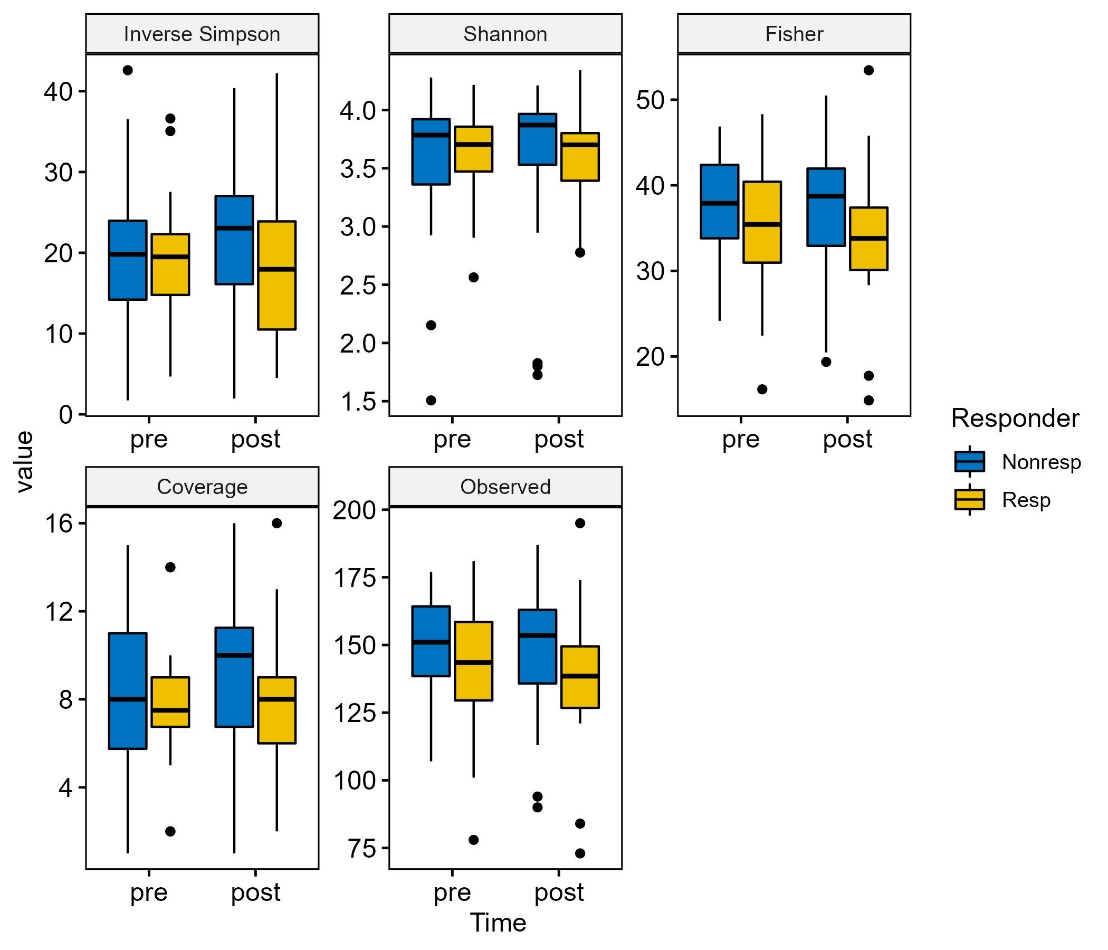


**Supplemental Fig. 3** Alpha diversity indices of responder (n=20) and non-responder (n=23) before and after 12 weeks of Lactobacillus plantarum intake


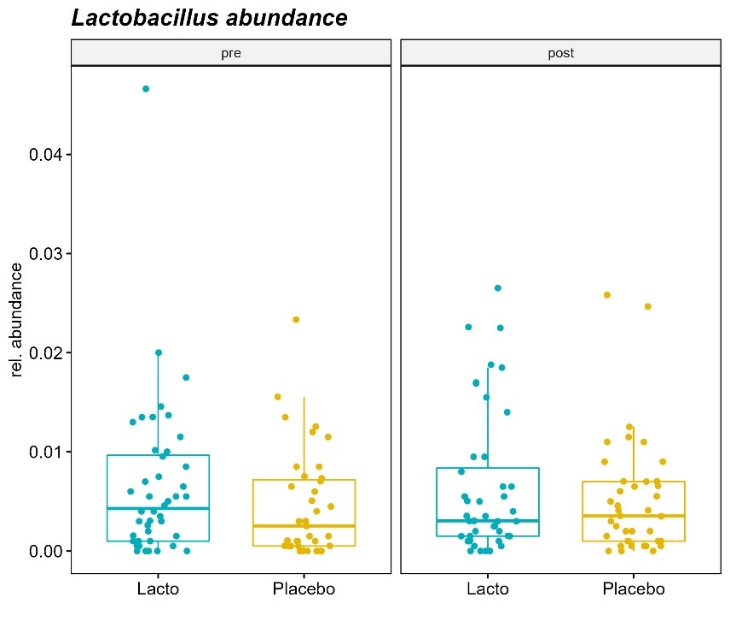


**Supplemental Fig. 4** Relative fecal abundance of Lactobacillus in the Lacto (n=43) and placebo (n=43) group before and after the intervention


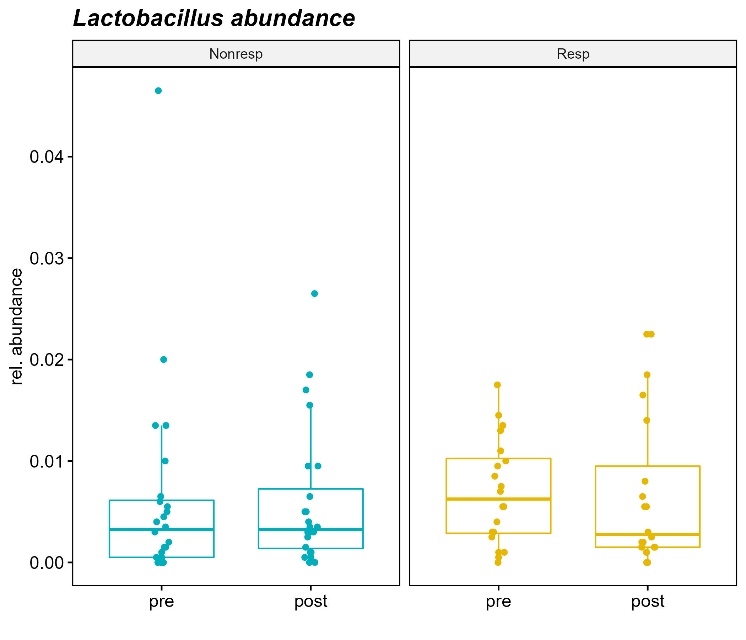


**Supplemental Fig. 5** Relative fecal abundance of *Lactobacillus* in responder (n=20) and non-responder (n=23) before and after the intervention

**Supplemental Table 3** Association between microbial taxa (genus level), responder status and clinical variables in the Lacto group (n=43).

| **Taxa (Genus level)** | **Value** | **coefficient** | **P-Value** | **Q-Value** |
| --- | --- | --- | --- | --- |
| *Roseburia* | Responder | 1.01 | >0.001 | 0.050 |
| *Oscillibacter* | Responder | -1.36 | >0.001 | 0.050 |
| *Oscillibacter* | ∆LDL-C | 0.66 | 0.001 | 0.057 |
| *Oscillibacter* | ∆total cholesterol | 0.55 | 0.008 | 0.212 |
| *Roseburia* | ∆total cholesterol | -0.38 | 0.016 | 0.237 |
| Coef= model coefficient value for the OTU, q-value=FFR-corrected significance of the association based on the whole dataset, ∆ change between post –pre timepoints, LDL-C, low-density lipoprotein cholesterol | | | | |
